# Development of semi-defined rice straw-based medium for butanol production and its kinetic study

**DOI:** 10.1007/s13205-013-0120-x

**Published:** 2013-03-09

**Authors:** Amrita Ranjan, Rahul Mayank, Vijayanand S. Moholkar

**Affiliations:** 1 Centre for Energy, Indian Institute of Technology Guwahati, Guwahati, 781 039 Assam India; 2 Department of Chemical Engineering, Indian Institute of Technology Guwahati, Guwahati, 781 039 Assam India

**Keywords:** Rice straw hydrolysate, Optimization, Media development, Biobutanol, Taguchi DOE method, ABE fermentation

## Abstract

Rice straw is one of the potential economic feedstock for biobutanol production through ABE fermentation. However, the rice straw hydrolysate-based fermentation medium needs to be supported with nutritional elements. In this study, an attempt is made to optimize the rice straw hydrolysate-based fermentation medium employing *Clostridium acetobutylicum* MTCC 481 using Taguchi design of experiments (DOE) statistical model. Initially, a set of 12 nutrient components viz. MgNO_3_·6H_2_O, FeNO_3_, NH_4_NO_3_, yeast extract, PABA, biotin, PABA + biotin mixture, CaCl_2_, KCl, NaCl, MgSO_4_ and CH_3_COONa were screened through classical (one-variable-at-a-time) method. Based on the results, four components (PABA, yeast extract, MgSO_4_ and CH_3_COONa) were found to have significant impact, and were further subjected to statistical optimization through Taguchi DOE method. These experiments revealed that RSH supported with 3 g L^−1^ of yeast extract and 4 mg L^−1^ PABA to RSH was the most optimum fermentation medium. Experiments using 2 L bioreactor with this optimum fermentation medium showed nearly complete utilization of soluble sugars with the production of 8.7 g L^−1^ of total solvents and 6 g L^−1^ of butanol. The experimental data were fitted to kinetic models reported in the literature to determine the kinetic parameters of the fermentation process. An interesting result was revealed from this analysis that the under optimized fermentation medium, the kinetic parameters for both shake flask and bioreactor level were similar. This essentially means that effect of scale of operation is rendered insignificant when fermentation medium is under optimum conditions.

**Electronic supplementary material:**

The online version of this article (doi:10.1007/s13205-013-0120-x) contains supplementary material, which is available to authorized users.

## Introduction

Anaerobic clostridial acetone–butanol–ethanol (ABE) fermentation from variety of feedstocks is one of the oldest and the largest biotechnology processes implemented till mid-last century, mainly for production of acetone. The other products from this process are butanol and ethanol, which are potential alcoholic biofuels. In the recent years, fast depletion of fossil fuels, highly fluctuating prices of crude oil and concerns of greenhouse gas emissions and global warming have revived interests of scientific community in the ABE fermentation. Butanol has emerged as a potential alternate liquid transportation fuel, and it has distinct merits such as high energy density (almost same as that of gasoline), low vapour pressure, low heat of vaporization, high hydrophobicity (non-polar character), and non-corrosive nature. Due to these properties, storage and transport of biobutanol are relatively much easier than other liquid biofuels. In addition, butanol can be blended with gasoline in any percentage (as against only 10–20 % for biodiesel and ethanol), or can be directly used in the existing internal combustion engines with nearly no modifications required (Ranjan and Moholkar 2011). The major impediments in economic production of butanol through ABE fermentation are high substrate cost, low yield and high cost of product recovery. Significant research has been dedicated in last two decades for exploration of variety of alternate cheap renewable feedstocks for ABE fermentation such as bagasse, wheat straw, wheat bran, corn fibre and other agriculture waste. A comprehensive review of literature in this area has been recently published by Ranjan and Moholkar (2012).

Abundantly available biomass in the form of agro-residues and forest-residues can be effectively utilized for the fermentation process (Jin and Chen 2007). However, there are inherent difficulties in direct utilization of these biomasses for fermentation as lignocellulose (comprised of cellulose, hemicellulose and lignin) is the main constituent of these biomasses (Liu et al. 2012; Poornejada et al. 2013). Natural lignocellulosic biomass is resistant to hydrolysis because of the crystallinity of cellulose, protection of cellulose by lignin, cellulose sheathing by hemicelluloses and the limited accessible surface area of the biomass matrix. Pre-treatment of biomass via physical, chemical, enzymatic or microbial route is critical to collapse the close knit structure of lignocellulosic biomass, thus providing hydrolysis agents with a more direct access to the feedstock (Yu et al. 2009). Various modes of pre-treatment have been attempted for hydrolysing lignocellulosic biomass for release of fermentable sugars, such as acid hydrolysis, alkaline hydrolysis, steam treatment or steam explosion, microwave treatment, enzymatic hydrolysis, etc. (Shukla and Cheryan 2001; Roberto et al. 2003; Xu and Hanna 2010; Li et al. 2011a; Jiang et al. 2011; Dagnino et al. 2013; Singh and Bishnoi 2012). The general process for alcoholic biofuel production using lignocellulosic biomass as feedstock involves pre-treatment and hydrolysis of feedstock followed by fermentation (Buranov and Mazza 2008; Miao et al. 2011; Monteil-Rivera et al. 2013). Our research mainly focuses on utilization of rice straw as potential feedstock for biobutanol production.

Pretreated rice straw hydrolysate (RSH) being nutritionally deprived does not act as complete medium for solvent production using clostridial strain. For successful ABE fermentation, the rice straw hydrolysate needs to be supplemented with additional components or nutritional factors comprising of vitamins, minerals and salts (collectively called as fermentation medium) that support the growth and metabolism of clostridial cultures, which eventually manifests in enhanced solvent production (Dabrock et al. 1992; Moon et al. 2011). These vitamins, minerals and other nutrients act as inducers and/or co-factors for enzymes that participate in the metabolism leading to solvent production. The literature reports several studies on growth media (i.e. supplementary components in the fermentation medium that support growth) for clostridial cultures (Soni et al. 1987; Leclerc et al. 1998; Somda et al. 2011). These growth media comprise of numerous components such as amino acids, trace metals, vitamins, growth factors, carbon sources, etc. Some of these components could be critical for cell growth or productivity, while others may be toxic at certain levels, and many may be involved in complex interactions within the cell. Li et al. (2011b) have reported that the adequate addition of yeast extract in fermentation medium could promote phase shift (acidogenesis–solventogenesis) by increasing gene transcription to 16-fold, and indirectly enhance butanol synthesis through accelerating the accumulation of histidine and aspartic acid families. Similarly, Haapalainen et al. (2007) have reported the importance of potassium and chloride ion for the activation of the enzyme thiolase, which play a key role in clostridial metabolism for the production of butanol. Dehydrogenases (butanol dehydrogenase, ethanol dehydrogenase, alcohol dehydrogenases, etc.) are the important enzymes of clostridial metabolic pathway leading to formation of ethanol, butanol and reversible oxidation of ethanol to acetaldehyde with the concomitant reduction of NAD. The major requisites for the normal functioning of these enzymes include Zn^2+^, Fe and other vitamins and minerals, which can be fulfilled by addition of yeast extract, PABA, Zn salts, iron salts, etc. to the fermentation medium (Walter et al. 1992). In order to find the best composition of these components in the medium for fermentation aimed at maximization of production of butanol, an optimization study is essential. This paper addresses this important issue using Taguchi statistical experimental design method.

This article is an attempt to develop a complete rice straw-based fermentation medium suitable for enhanced butanol production via clostridial fermentation. In our approach, we first studied the effect of selected nutritional factors reported in the literature on the production of butanol by *Clostridium acetobutylicum* microbial type culture collection (MTCC) 481 strain using the classical ‘one-variable-at-a-time’ method. The significance of the effect of these nutritional factors on butanol production was then analysed using a statistical Taguchi experimental design (Dasu et al. 2003; Chang et al. 2006). Finally, the solvent production in the optimized fermentation medium was tested first on shake flask scale, and later in a 2 L stirred tank bioreactor operated in batch mode without pH control.

## Materials and methods

### Micro-organism, culture revival and maintenance

Lyophilized cells of *C. acetobutylicum* MTCC 481 were procured from MTCC, Institute of Microbial Technology (Chandigarh, India). The cells were revived anaerobically inside an anaerobic culture bag system (Himedia) in reinforced clostridial agar (RCA) and reinforced clostridial medium (RCM: Broth) culture media at 37 °C. The inoculums were prepared in RCM containing following components (with concentration mentioned in g L^−1^): glucose 5.0; yeast extract 3.0; starch 1.0; beef extract 10.0; peptone 10.0; sodium chloride 5.0; sodium acetate 3.0; agar 0.5 and cysteine hydrochloride 0.5. The pH of medium was 6.8 ± 0.2. 100 mL of media was autoclaved at 121 °C, 15 lb pressure and inoculated in a custom fabricated 250 mL screw capped Erlenmeyer flask. In addition, cooked meat medium (CMM) was also used for the maintenance of clostridia. Anaerobic condition in broth culture was maintained by adding 0.05 % of cysteine hydrochloride and regular sparging of nitrogen to fermentation broth. All chemicals were of analytical grade procured either from Merck (Germany), Sigma Aldrich (Germany) or Himedia (India). The revived cells were maintained on RCM agar plates and slants at 4 °C, and were used as a stock. The cells were sub-cultured every month.

### Preparation of rice straw hydrolysate (RSH)

After initial comminution process (washing, drying, cutting, grinding) of rice straw, an aqueous mixture of 5 % w/v RS was prepared. This mixture was hydrolysed using 1 % H_2_SO_4_ and was autoclaved for 15 min at 15 lb steam pressure. This RSH was further stirred at 60 °C and 200 rpm resulting in smooth slurry. This was then filtered using sterile muslin cloth and the supernatant was used as fermentation broth. This was further supplemented with nutrients as per the methodology of optimization as mentioned later in the text. All chemicals used as supplements (MgNO_3_·6H_2_O, FeNO_3_, NH_4_NO_3_, yeast extract, PABA (*p*-aminobenzoic acid), biotin, CaCl_2_·2H_2_O, KCl, NaCl, MgSO_4_·7H_2_O and CH_3_COONa) were of analytical grade and were procured from either Merck or Himedia.

### Fermentation conditions

The initial screening of the media constituents was done using conventional method of one-variable-at-a-time approach. *C*. *acetobutylicum* MTCC 481 strain was cultured in acidified RSH medium. 12 sets of fermentation experiments supplemented with following single additives (in g L^−^) were carried out: MgNO_3_·6H_2_O 3.0; FeNO_3_ 3.0; NH_4_NO_3_ 3.0; yeast extract 3.0; PABA 0.02; biotin 0.01; PABA and biotin 0.02 and 0.01; CaCl_2_·2H_2_O 0.02; KCl 0.5; NaCl 0.01; MgSO_4_·7H_2_O 0.2 and CH_3_COONa 3.0. These media components and their concentrations were selected on the basis of previous literature (Hans 1975; Leclerc et al. 1998; Somda et al. 2011; Soni et al. 1987 Li et al. 2011b). Table 1 depicts the solvent (acetone, butanol and ethanol) production in above 12 sets of experiments. Out of these 12 components, four components, viz. yeast extract, PABA, MgSO_4_·7H_2_O and CH_3_COONa, showed noteworthy potential for butanol production and were short-listed for further Taguchi experimental design, the details of which are described in next section. The optimization studies were carried out in custom fabricated 250 mL anaerobic Erlenmeyer flasks (picture supplied as supplementary material) with bottom port for sample withdrawal and nitrogen sparging in order to minimize oxygen contamination and maintain strict anaerobic conditions. Each flask contained fermentation medium with desired composition (i.e. mixture of 100 mL of acidified RSH and nutritional factors), and was inoculated with 2 % of 18 h old, *C*. *acetobutylicum* MTCC 481 culture. All flasks were sparged with nitrogen at the start and after every 24 h of fermentation to maintain anaerobic conditions. These flasks were kept in an incubator shaker (make: Scigenics Biotech, model: Orbitek) operating at 200 rpm and 37 °C. The samples of fermentation broth were withdrawn after every 2 days up to a period of 10 days. The initial pH of each set of acidified RSH-based medium was 1.0 ± 0.2.

**Table 1:** Solvent production from pretreated RSH supplemented with single variable (nutrient) at time

S. no.	Components	Acetone (g L^−1^)	Butanol (g L^−1^)	Ethanol (g L^−1^)
1	Magnesium nitrate hexahydrate	0.3	–	–
2	Ferric nitrate	–	–	–
3	Ammonium nitrate	–	–	–
4	Yeast extract	3.61	0.158	–
5	Paraaminobenzoic acid	3.71	0.167	0.02
6	Biotin	3.62	0.026	–
7	PABA + biotin	4.01	0.043	–
8	Calcium chloride	–	–	–
9	Potassium chloride	–	–	–
10	Sodium chloride	3.93	–	0.01
11	Magnesium sulfate	3.69	0.182	0.04
12	Sodium acetate	3.58	0.055	–

### Taguchi’s orthogonal array

A well-known experimental design technique, namely L8 orthogonal array design (Oskouie et al. 2007; Wu et al. 2010) was employed to study the effect of four parameters, viz. yeast extract, PABA, sodium acetate and MgSO_4_, on the butanol production. Essentially, the design consisted of a total of eight experiments with the four parameters, each having two levels, i.e. L8 (2^4^). Table 2 presents the combinations of experimental conditions adopted as per the design, along with butanol concentration as the observed response in each run. All experiments in this study were performed in custom fabricated 250 mL Erlenmeyer flasks with combination of parameters according to the experimental design (Table 2). To study the bio-butanol production in the batch shake flasks, 100 mL of optimized RSH medium was inoculated with seed culture and agitated in an incubator shaker (make: Scigenics Biotech, model: Orbitek) for 10 days at 37 °C and 200 rpm. Samples were taken periodically for the analyses of the residual sugar and ABE solvent production in the fermentation medium. All batch shake flask experiments were conducted in triplicate. The optimal conditions with respect to the experimental factors or parameters (which are essentially the nutritional factors) tested have been determined on the basis of average of signal to noise ratio (*S*/*N*) for the factor or parameter at each factor level (Table 3). Butanol production by *C. acetobutylicum* MTCC 481 was considered as a desired variable, and a higher concentration was preferred. In each experimental run, the response was recorded as the butanol production and corresponding *S*/*N* ratio was calculated using Eq. 1 with an overall objective of estimating the effects of various parameters on solvent production, where a large*S*/*N* ratio is preferred (Sanjari et al. 2009; Daverey and Pakshirajan 2010).1where *Y* is the response and *n* is the number of experimental runs. The statistical significance of each factor was determined using analysis of variance (ANOVA). Finally, the optimum conditions for butanol production by *C. acetobutylicum* MTCC 481 were again determined using ANOVA performed using MINITAB^®^ Release 15.1, PA, USA (trial version).

**Table 2:** Taguchi design matrix and corresponding butanol production by *Clostridium acetobutylicum* (MTCC 481) in shake flask

Experimental run no.	Parameters (factor)				Butanol (g L^−1^)	*S*/*N* ratio (dB)
	Yeast extract (g L^−1^)	PABA (mg L^−1^)	Sodium acetate (g L^−1^)	MgSO_4_ (g L^−1^)		
1	1 (3)	1 (2)	1 (3)	1 (0.2)	2.14	6.13
2	1 (3)	1 (2)	2 (5)	2 (0.5)	2.38	7.15
3	1 (3)	2 (4)	1 (3)	2 (0.5)	5.5	14.61
4	1 (3)	2 (4)	2 (5)	1 (0.2)	3.28	10.02
5	2 (5)	1 (2)	1 (3)	2 (0.5)	0.99	−0.62
6	2 (5)	1 (2)	2 (5)	1 (0.2)	1.5	3.27
7	2 (5)	2 (4)	1 (3)	1 (0.2)	1.45	2.41
8	2 (5)	2 (4)	2 (5)	2 (0.5)	1.44	2.18

**Table 3:** Value of average *S*/*N* ratio through Taguchi analysis of factors affecting butanol production by *Clostridium acetobutylicum*

Level	Factors			
	Yeast extract (g L^−1^)	PABA (mg L^−1^)	Sodium acetate (g L^−1^)	MgSO_4_ (g L^−1^)
1	9.472	2.4869	4.1367	5.4554
2	0.3142	7.3025	5.6527	4.3340
Delta*	9.1611	4.8157	1.5161	1.1213
Rank	1	2	3	4

* Degree of freedom

### Validation of results

Acetone–butanol–ethanol fermentation with optimized media components were validated in 250 mL custom fabricated Erlenmeyer flasks using the Taguchi reported optimized protocol, mentioned in previous section. Thereafter, the developed RSH-based fermentation media were tested in a 2 L microprocessor-controlled bioreactor (Zenith, India) with 1 L of developed RSH medium and 2 % v/v of clostridial inoculum. The temperature was controlled at 37 °C, and 99.98 % pure nitrogen was sparged at a rate of 0.1 vvm. The initial pH of the media and the agitation rate were set at 1.0 ± 0.2 and 200 rpm, respectively. The samples of fermentation broth were withdrawn after every 48 h up to a period of 10 days for analysis. The experiments in both shake flask and bioreactor were carried out in duplicate.

### Analysis

The optical density of the cells in the broth was measured using UV–vis spectrophotometer (Thermo Fischer) with absorbance at 600 nm after appropriate dilution in water. Glucose was analysed using Glucose (GO) assay kit procured from Sigma Aldrich, USA (GAGO20–1KT). Total sugar analysis was done by anthrone test as directed by Hedge and Hofreiter (1962). Quantification of reducing sugar has been done by using dinitrosalicylic acid (DNS) method proposed by Miller (1972). Samples were filtered with 0.2 μm filter and diluted appropriately for the qualitative and quantitative determination of the solvents and sugars.

**Fig. 1:**
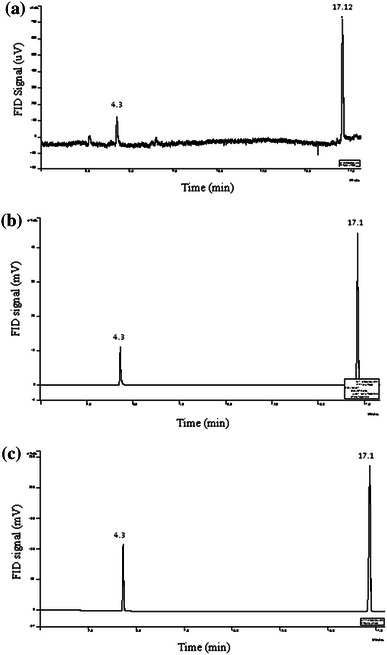
Gas chromatograms of fermentation broth for reactor scale experiments (employing optimized rice straw hydrolysate based medium) at various stages of fermentation: **a** 2 days, **b** 4 days, **c** 10 days

Solvent production in the fermentation broth was monitored on a gas chromatograph (Varian) using a CP Wax 52 CB (250 × 0.25 × 0.39 mm) capillary column and a flame ionization detector. The injector and detector temperatures were 230 and 250 °C, respectively. The oven temperature was programmed from 45 to 100 °C with an increment of 3 °C min^−1^, and after 100 °C, an increment of 5 °C min^−1^ up to 200 °C. Gas chromatograph was used to quantify acetone, butanol and ethanol during the fermentation of RSH. This was achieved by plotting standard calibration curves (peak area vs. concentration) using GC grade acetone, butanol and ethanol procured from Sigma Aldrich. *R*^2^ values for standard curve of acetone, butanol and ethanol were observed to be 0.98, 0.99 and 0.98, respectively. Samples were injected at regular intervals. Retention time for acetone was noted to be 4.3 min, while for butanol and ethanol 17.11 and 7.23 min, respectively. Representative chromatograms of the fermentation broth after 2, 4 and 10 days of fermentation are depicted in Fig. 1.

## Results and discussion

Preliminary conventional type screening experiments helped in determining the influence of supplemental nutritional factors to RSH on solvent production. However, in the present paper, we have emphasized on butanol production as the response of all experiments, rather than total solvent concentration, as butanol is the desired target product. Table 1 shows the results obtained after implementing one-variable-at-a-time approach. It could be inferred from Table 1 that among the 12 supplemental nutritional factors, MgNO_3_·6H_2_O, FeNO_3_, NH_4_NO_3_, biotin, CaCl_2_·2H_2_O, KCl, NaCl, PABA and biotin had an insignificant impact on butanol production, while PABA, yeast extract, MgSO_4_·7H_2_O and sodium acetate had a significant impact on the products yield. The results obtained with supplemental MgNO_3_·6H_2_O, NH_4_NO_3_, biotin, NaCl and (PABA + biotin) showed production of acetone and ethanol, while no solvent (ABE) production was observed when RSH was supplemented with FeNO_3_, CaCl_2_·2H_2_O and KCl. These results are in agreement with the work reported by Li et al. (2011b). According to Li et al. (2011b), yeast extract contains abundant of vitamins, minerals and amino acids, which are necessary for cell growth and synthesis of various enzymes. RSH is a deficient medium for nitrogen, vitamins and minerals and other trace elements, which act as cofactor for many enzymes. Yeast extract and PABA being a rich source of nitrogen as well as vitamins and minerals resulted in enhanced clostridial growth and metabolism, which has manifested consequently in enhanced solvent production (Leclerc et al. 1998; Soni et al. 1987). Sodium acetate, being a source of acetate which is also a product during acidogenesis phase (that is further converted to solvents during the subsequent solventogenesis) probably acted as an inducer of solventogenesis. MgSO_4_ (also known as epsom salt) is known for its effect on improvement of uptake of other nutrients, which results in enhancement of growth. Gawande et al. (1998), Somda et al. (2011), Birch and Walker (2000) and Pasternak et al. (2010) have also found Mg^2+^ to be essential for enzyme production and consequently alcohol release. Mori et al. (1985) showed that magnesium acts as activator of some enzymes, especially transferases and decarboxylases, which play an important role in biochemistry of alcohol production. Thus, the results of our preliminary or one-variable-at-a-time experiments were in good agreement with earlier study. Thus, the four factors, viz. yeast extract, PABA, MgSO_4_ and sodium acetate, were selected as main or significant factors, and were allowed to undergo further statistical optimization through Taguchi design of experiments (DOE) method.

### Taguchi analysis and ANOVA

**Fig. 2:**
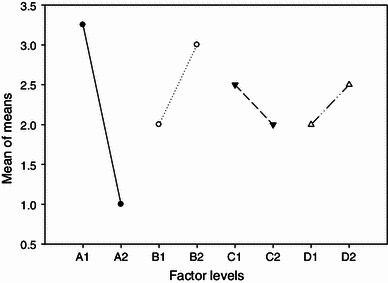
Main effects plot of mean for the process variables. *A1* and *A2* two levels of yeast extract, *B1* and *B2* two levels of PABA, *C1* and *C2* two levels of MgSO_4_, *D1* and *D2* two levels of sodium acetate

Table 2 depicts the final total concentration of alcohols formed with media comprising of different compositions of significant factors, i.e. yeast extract, PABA, MgSO_4_ and sodium acetate. The results given in Table 2 indicate that the production of solvents is a major function of media composition. Among all eight experimental runs, the highest total solvents (8.04 g L^−1^) and butanol (5.5 g L^−1^) concentration was attained in run 3. The percentage effect of each factor on butanol and total solvent production is given in Table 3. Out of four factors examined, yeast extract and PABA had major effect on butanol production, as indicated by their high delta *S*/*N* value of 9.16 and 4.82 db, respectively. In Taguchi statistical design, *S*/*N* ratio is an important parameter for identifying optimal conditions for the process. A high *S*/*N* ratio indicates higher significance of the factor. Based on this logic, the order of effect of various factors on total alcohol production was determined as yeast extract > PABA > CH_3_COONa > MgSO_4_·7H_2_O. The percent individual contribution of these four factors to the main effect mean (or delta *S*/*N*) was in order: 55.14 % (yeast extract) > 28.99 % (PABA) > 9.13 % (sodium acetate) > 6.75 % (magnesium sulphate), indicating yeast extract and PABA to be the factors of greater (relative) significance, and sodium acetate and magnesium sulphate as relatively insignificant factors with lower percent contribution for butanol production (<10 %). Figure 2 displays the main effect of means of process variables, viz. yeast extract, PABA, MgSO_4_·7H_2_O and sodium acetate. The total solvent production was higher at level 1 for factors yeast extract and MgSO_4_, while factors PABA and sodium acetate produced more solvents at level 2. The comparatively insignificant effect of sodium acetate and MgSO_4_ on production of butanol can be explained in terms of understanding clostridial metabolism, where cells themselves produce acetate by utilizing carbon source from the medium, and hence, additional supplementation of acetate resulted in no net gain in terms of butanol production (Gheshlagi et al. 2009). Similarly, as stated earlier, Mg^2+^ from MgSO_4_ is required for proper functioning of enzymes that are key components of xylose metabolism. As *C. acetobutylicum* preferably utilizes glucose over pentose (Grimmler et al. 2010), Mg^2+^ requirement of cells become insignificant.

ANOVA was done to quantify the variation in product formation caused due to each factor, and also to determine as whether the lower or higher value of a factor is essential for preferred result, i.e. higher butanol production. Table 4 depicts the results of ANOVA for all factors. The ANOVA results corroborate the results based on *S*/*N* ratio in which the significant factors for butanol production (which had more than 10 % contribution to the main effect with *p* value <0.005, *F* value >5, and higher confidence level) were yeast extract and PABA, while sodium acetate and MgSO_4_ were insignificant factors, as indicated by *p* > 0.005 and *F* < 5 (Oskouie et al. 2007; Venil and Lakshmanaperumalsamy 2009). The ANOVA of butanol production (Table 4) had a model sum of squares (SS), mean of squares (MS), and *F* value of 9.61, 2.12 and 29.56, respectively. The model obtained from ANOVA had *R*^2^ (multiple regression coefficient) value of 0.88, which is indicative of the robustness of the model in which it can explain ~88 % variation in the response. Thus, optimized RSH-based fermentation medium for butanol production consisted of stress-assisted acid-treated RS supplemented with 3.0 g L^−1^ yeast extract and 4.0 mg L^−1^ PABA.

**Table 4:** Analysis of variance (ANOVA) of butanol production

Source	DF*	SS^#^	MS	*F* ratio (*F*)	*p* value (*p*)	Confidence level (%)	Percent contribution
Yeast extract	1	5.9004	5.9004	98.33	<0.005	96.09	55.14
PABA	1	2.036	2.036	14.45	<0.005	78.32	28.985
Sodium acetate	1	0.2076	0.2076	3.6	>0.005	58.32	9.125
MgSO_4_	1	0.3541	0.3541	1.89	>0.005	68.96	6.749
Residual error	3	1.1118	0.2779				
Model	7	9.6099	2.12				100

* Degree of freedom, ^#^ Sum of squares

### Prediction of butanol and total solvents production with optimized medium

To predict the butanol production (denoted by *Y*_opt_) for the optimized fermentation, medium mentioned above the following formula was used:2

Here,  denotes the average butanol production in all trial results, while *A*_1_ and *A*_2_ are the average butanol productions at two levels of yeast extract, while *B*_1_ and *B*_2_ are average butanol productions for the two levels of PABA (Sirisansaneeyakul et al. 2007; Sanjari et al. 2009). Using above formula, the predicted values of butanol and total solvent productions for the optimized fermentation medium were 5.5 and 8.04 g L^−1^, respectively.

### Validation and comparison of results

**Fig. 3:**
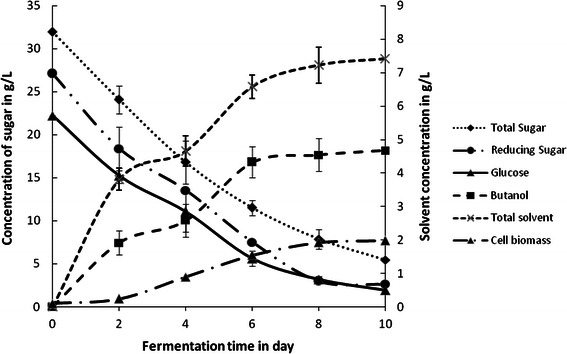
Time history of solvent production and sugar utilization in the shake flask experiment with optimized fermentation medium

**Fig. 4:**
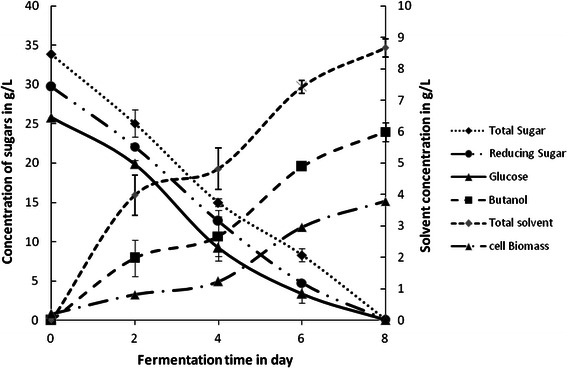
Time history of solvent production and sugar utilization in 2 L bioreactor experiment with optimized fermentation medium

Confirmatory experiments to assess butanol and total solvent production with optimized fermentation medium were carried out in duplicate, at both shake flask level as well as in a 2 L bioreactor (make: Zenith, India). Both solvent and butanol concentration were substantially similar to the predicted optimal values (mentioned in previous section). Moreover, these results were quite comparable to the values previously attained in the experiment trial 3, for which the yeast extract and PABA concentrations were 3 g L^−1^ and 4 mg L^−1^, respectively (Table 3). Shake flask confirmatory experiments with optimized nutritional conditions at 37 °C, pH 1.0 and 200 rpm for 10 days resulted in production of 7.4 g L^−1^ of total solvents, and 4.67 g L^−1^ of butanol. The time history of solvent production and sugar utilization by *C. acetobutylicum* MTCC 481 at shake flask scale and reactor scale is depicted in Figs. 3 and 4, respectively. It was observed that clostridia were able to utilize most of the sugar present in RSH-based fermentation medium. Nearly 91.4 % glucose, 90 % reducing sugar and 83 % total sugar were utilized during 10 days of fermentation. These values of high sugar utilization with concurrent production of solvents corroborate the suitability of optimized RSH-based fermentation medium for *C. acetobutylicum.* With scaling up of this processes in a 2 L bioreactor (or fermentor), results were more encouraging. Nearly 6.0 g L^−1^ of butanol and 8.7 g L^−1^ of total solvent were produced with almost complete utilization of all sugar in only 8 days. These values are in good agreement with the predicted results by Taguchi method. The reduction in fermentation time, elevated solvent production and higher substrate utilization profile in the bioreactor could be attributed to control of physical parameters with greater precision, in addition to maintenance of ideal anaerobic conditions in the microprocessor-based control system in the bioreactor, as compared to the shake flask experiments, where the control of process parameters and anaerobic conditions could have been less precise.

Table 5 presents the comparison of results of the present study with those reported in earlier studies using cheap alternate substrates. It can be perceived from the results presented in Table 5 that rice straw is indeed a potential substrate giving results that are at par with other agro-residues like wheat straw and corn fiber. As compared to other substrates like cassava pulp, algal biomass and DDGS, the butanol yield is much higher with rice straw. An interesting result is that under similar conditions as used in this work (i.e. acid hydrolysis of substrate prior to fermentation), the butanol production from wheat and rice straw is almost similar, as evident from the work of Qureshi et al. (2008b). However, if acid treatment of substrate is also supplemented with a suitable enzyme that helps release of more sugar due to breakage of β-glycosidic linkages of cellulose, greater solvent productivity is seen as evident from results of Qureshi et al. (2008b).

**Table 5:** Comparative evaluation of butanol production with rice straw and other alternate substrates

References	Microorganism	Substrate	Butanol titre (g L^−1^)
This paper	*C. acetobutylicum* MTCC 481	Rice straw	6.0
Virunanon et al. (2013)	*C. butyricum*, *S. cerevisiae*	Cassava pulp	2.51
He and Chen (2012)	*C. acetobutylicum* ATCC 824	Corn stover + glucose/xylose	9.64
Guo et al. (2012)	*C. beijerinckii* IB4	Corn fiber	6.8
Qureshi et al. (2008a)	*C. beijerinckii* BA101	Corn fiber	5.8
Efremenko et al. (2012)	Immobilized *C. acetobutylicum* B1787	*Arthrospira platensis* (algae)	0.43
Wang et al. (2013)	*C. beijerinckii* BA 101	Distillers dried grains with solubles (DDGS)	3.62
Qureshi et al. (2008b)	*C. beijerinckii* P260	Wheat straw (under different processes^a^)	Process I 6.05 Process II 8.09 Process III 7.4 Process IV 5.7

^a^*Process I* dilute acid hydrolysed wheat straw; *Process II* acid + enzyme hydrolysis of wheat straw; *Process III* acid + enzyme treated wheat straw followed simultaneous saccharification and fermentation; *Process IV* acid + enzyme treated wheat straw with solvent removal by gas stripping

### Kinetic analysis of solvent production, biomass growth and utilization of sugars under optimized nutrient conditions

The time history of butanol production, clostridial biomass growth and utilization of sugar using optimized fermentation medium in shake flask experiments and also experiments using microprocessor controlled bioreactor (make: Zenith) have been illustrated in Figs. 3 and 4. It can be seen from Figs. 3 and 4 as compared to the shake flask experiments, the fermentation in scaled-up bioreactor resulted in reduced lag phase with an exponential phase of up to 36 h, followed by an extended stationary phase of 96 h, during which production of solvents occurred. This observation is consistent with our earlier paper on the clostridial biomass growth and butanol production (Ranjan and Moholkar 2011). Solvent production profiles in shake flask and bioreactor experiments showed that the solvent production (including butanol) occurred till 192 h of fermentation cycle. The concentration profiles of sugars (total sugar, reducing sugar and glucose) in the medium for both shake flask and bioreactor scale experiments showed that they get readily utilized by clostridial cells. Shake flask experiment resulted in utilization of 91 % of total sugar, 95 % of reducing sugar and 96 % of glucose at the end of fermentation cycle, while reactor scale optimized run resulted in complete utilization of all sugar displaying the enhanced fermentation efficiency at reactor scale.

**Fig. 5:**
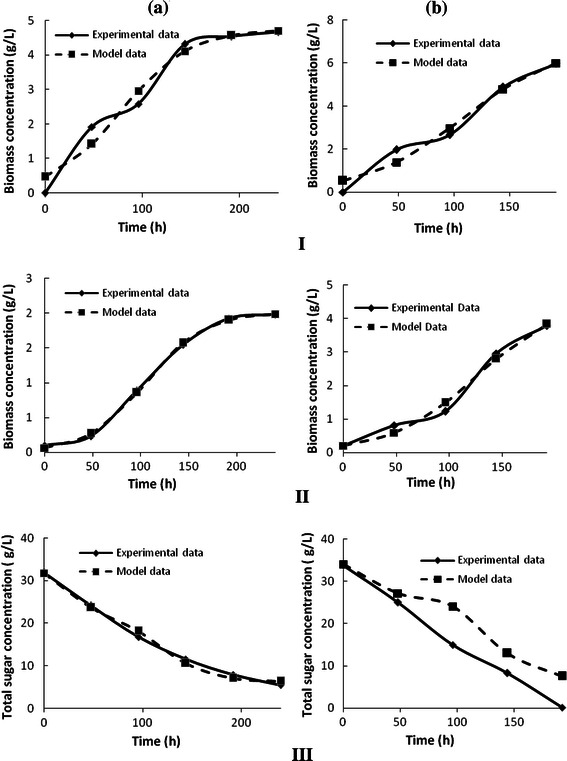
Verification of experimental data through comparative study with model data. **a** Flask scale optimized process. **b** Reactor scale optimized process. *I)* Comparative study of experimental and model data for clostridial butanol production during ABE fermentation. *II)* Comparative study of experimental and model data for clostridial biomass growth during ABE fermentation. *III)* Comparative study of experimental and model data for total sugar utilization in terms of total solvents and biomass

Further, to estimate the bio-kinetic constants involved in the process, models based on Monod kinetics reported in the literature (Mercier et al. 1992; Rodrigues et al. 2006) were fitted to the experimental data of butanol production, clostridial biomass growth and utilization of sugar. Table 6 presents the kinetic models applied in this study along with the calculated kinetic parameters by fitting the experimental data using Matlab 7.12. These models are essentially unstructured logistic models originally proposed by Mercier et al. (1992) for describing the kinetics of biomass growth, substrate consumption and product accumulation. These models have also been applied earlier by Daverey and Pakshirajan (2010) and Rodrigues et al. (2006) to explain the sophorolipid and biosurfactant production kinetics, in their respective studies. The estimated kinetic parameters values obtained from these models for both shake flask and bioreactor scale experiments are listed in Table 6. Figure 5 shows trends of experimental results (i.e. butanol production, biomass growth and sugar utilization) along with the trends predicted by the models. The regression coefficients (*R*^2^) for the models fitted to the experimental data are ≥0.9, which is indicative that the model prediction is in good agreement with the experimental data. Further, goodness of the model fitting is also determined in terms of lower values of the root-mean-square deviation (RMSD) or root-mean-square error (RMSE), and these values have been listed in Table 6. The values of *P*_max_, i.e. maximum product concentration predicted by the model for both shake flask (4.752 g L^−1^) and bioreactor (5.03 g L^−1^) match well with the experimentally observed values of 4.67 and 6 g L^−1^, respectively, mentioned in previous section. For both shake flask and bioreactor scale experiments, the specific growth rate of clostridial cultures has been quite low (*μ* ≈ 0.03 h^−1^), which indicates that optimized RSH is a suitable fermentation medium, where the cell utilizes most of its energy for product formation rather than cellular growth. From the kinetic parameter values presented in the Table 6, an interesting observation could be made that the values of *P*_t_ and *μ* are practically same for both shake flask and bioreactor experiments. These parameters are characteristics of the kinetics of the fermentation process. Practically, similar values of *μ* and *P*_t_ for shake flask and bioreactor scale experiments using optimized fermentation medium indicate that the effect of scale of operation on the fermentation kinetics and product profile becomes insignificant when fermentation medium is under optimized conditions.

**Table 6:** Models applied for the estimation of biokinetic constants and kinetic parameters estimated by fitting the models to experimental data obtained for shake flask and reactor scale study

Component	Rate equations	Flask scale process^a^			Reactor scale process^a^		
		Kinetic parameter	Regression coefficient (*R*^2^)	Root mean square error (RMSE)	Kinetic parameter	Regression coefficient (*R*^2^)	RMSE
Butanol		*P*_0_ = 0.4692	0.9616	0.469	*P*_0_ = 0.5286	0.9005	0.749
		*P*_max_ = 4.752			*P*_max_ = 5.03		
		*P*_t_ = 0.0282			*P*_t_ = 0.0289		
Biomass		*X*_0_ = 0.06284	0.9977	0.040	*X*_0_ = 0.07351	0.9733	0.687
		*X*_max_ = 2.01			*X*_max_ = 3.996		
		*μ* = 0.03278			*μ* = 0.0351		
Total sugar		*S*_0_ = 31.08		1.212	*S*_0_ = 32.43	0.9588	3.127
					*Y*_P/M_ = 0.59		
		*Y*_P/M_ = 0.5234	0.9915		*Y*_X/M_ = 0.2041		
		*Y*_X/M_ = 0.1676					

^a^Under optimized conditions

## Conclusion

The results of this study reveal that nutritionally optimized RSH medium (with added yeast extract and PABA) could be a potential alternative feedstock for biobutanol production. Furthermore, our results also reveal that production of ABE solvents through fermentation of pretreated RSH by *C. acetobutylicum* MTCC 481 is a strong function of composition of the media. The optimization of fermentation medium in terms of supplemental nutritional factors by Taguchi design has identified key factors responsible for enhanced production of solvents with emphasis on butanol, which is a potential alternate liquid transportation fuel. The results of this study could give vital inputs for design of an economic and efficient RSH-based fermentation process.

## Electronic supplementary material

Below is the link to the electronic supplementary material.

Supplementary material 1 (DOC 50 kb)

## List of symbols

*μ*: Specific growth rate (h^−1^)
*P*: Product concentration (g dm^−3^)
*P*_0_: Initial product concentration (g dm^−3^)
*P*_max_: Maximum product concentration (g dm^−3^)
*P*_t_: Kinetic constant
*X*: Biomass concentration (g dm^−3^)
*X*_m_: Maximum biomass concentration (g dm^−3^)
*X*_0_: Biomass concentration (g dm^−3^)
*Y*_P/S_: Product yield on the utilized substrate
*Y*_X/S_: Biomass yield on the utilized substrate

## References

[CR1] Birch RM, Walker GM (2000). Influence of magnesium ions on heat shock and ethanol stress responses of*Saccharomyces cerevisiae*. Enzym Microb Technol.

[CR2] Buranov AU, Mazza G (2008). Lignin in straw of herbaceous crops. Ind Crop Prod.

[CR3] Chang MY, Tsai GJ, Houng JY (2006). Optimization of the medium composition for the submerged culture of *Ganoderma lucidum* by Taguchi array design and steepest ascent method. Enzym Microb Technol.

[CR4] Dabrock B, Bahl H, Gottschalk G (1992). Parameters affecting solvent production by *Clostridium pasteurianum*. Appl Environ Microbiol.

[CR5] Dagnino EP, Chamorro ER, Romano SD, Felissia FE, Area MC (2013). Optimization of the acid pretreatment of rice hulls to obtain fermentable sugars for bioethanol production. Ind Crop Prod.

[CR6] Dasu VV, Panda T, Chidambaram M (2003). Determination of significant parameters for improved griseofulvin production in a batch bioreactor by Taguchi’s method. Process Biochem.

[CR7] Daverey A, Pakshirajan K (2010). Kinetics of growth and enhanced sophorolipids production by*Candida bombicola* using a low-cost fermentative medium. Appl Biochem Biotech.

[CR8] Efremenko EN, Nikolskaya AB, Lyagin IV, Senko OV, Makhlis TA, Stepanov NA, Maslova OV, Mamedova F, Varfolomeev SD (2012). Production of biofuels from pretreated microalgae biomass by anaerobic fermentation with immobilized *Clostridium acetobutylicum* cells. Biores Technol.

[CR9] Gawande BN, Singh RK, Chauhan AK, Goel A, Patkar AY (1998). Optimization of cyclomaltodextrin glucanotransferase production from *Bacillus firmus*. Enzym Microb Technol.

[CR10] Gheshlagi R, Scharer JM, Moo-Young M, Chou CP (2009). Metabolic pathways of Clostridia for producing butanol. Biotechnol Adv.

[CR11] Grimmler C, Held C, Liebl W, Ehrenreich A (2010). Transcriptional analysis of catabolite repression in *Clostridium acetobutylicum* growing on mixtures of d-glucose and d-xylose. J Biotechnol.

[CR12] Guo T, Tang Y, Xi YL, He AY, Sun BJ, Wu H, Liang DF, Jiang M, Ouyang PK (2012). *Clostridium beijerinckii* mutant with high inhibitor-tolerance obtained by low-energy ion implantation. J Ind Microbiol Biotechnol.

[CR13] Haapalainen AM, Meriläinen G, Pirilä PL, Kondo N, Fukao T, Wierenga RK (2007). Crystallographic and kinetic studies of human mitochondrial acetoacetyl-CoA thiolase (T2): the importance of potassium and chloride ions for its structure and function. Biochemistry.

[CR14] Han YW (1975). Microbial fermentation of rice straw: nutritive composition and in vitro digestibility of the fermentation products. Appl Microbiol.

[CR15] He Q, Chen H (2012) Improved efficiency of butanol production by absorbed lignocellulose fermentation. J Biosci Bioeng. doi:10.1016/j.jbiosc.2012.09.017 10.1016/j.jbiosc.2012.09.017 23085417

[CR16] Hedge JE, Hofreiter BT (1962) Carbohydrate chemistry. In: Whistler RL, Miller JN (eds), Academic press, New York, p 17

[CR17] Jiang M, Zhao M, Zhou Z, Huang T, Chen X, Wang Y (2011). Isolation of cellulose with ionic liquid from steam exploded rice straw. Ind Crop Prod.

[CR18] Jin S, Chen H (2007). Near-infrared analysis of the chemical composition of rice straw. Ind Crop Prod.

[CR19] Leclerc M, Elfoul-Bensaid L, Bernalier A (1998). Effect of yeast extract on growth and metabolism of H2-utilizing acetogenic bacteria from the human colon. Curr Microbiol.

[CR20] Li X, Cai Z, Winandyc JE, Bastad AH (2011). Effect of oxalic acid and steam pretreatment on the primary properties of UF-bonded rice straw particleboards. Ind Crop Prod.

[CR21] Li M, Liao X, Zhang D, Du G, Chen J (2011). Yeast extract promotes cell growth and induces production of polyvinyl alcohol-degrading enzymes. Enzym Res ID.

[CR22] Liu X, Lu M, Ai N, Yu F, Ji J (2012). Kinetic model analysis of dilute sulfuric acid-catalyzed hemicellulose hydrolysis in sweet sorghum bagasse for xylose production. Ind Crop Prod.

[CR23] Mercier P, Yerushalmi L, Rouleau D, Dochain D (1992). Kinetics of lactic acid fermentation on glucose and corn by *Lactobacillus amylophilus*. J Chem Tech Biotech.

[CR24] Miao Z, Grift TE, Hansen AE, Ting KC (2011). Energy requirement for comminution of biomass in relation to particle physical properties. Ind Crop Prod.

[CR25] Miller GL (1972). Use of dinitrosalicylic acid reagent for determination of reducing sugar. Anal Chem.

[CR26] Monteil-Rivera F, Phuong M, Ye M, Halasz A, Hawari J (2013). Isolation and characterization of herbaceous lignins for applications in biomaterials. Ind Crop Prod.

[CR27] Moon C, Lee CH, Sang BI, Um Y (2011). Optimization of medium compositions favoring butanol and 1,3-propanediol production from glycerol by *Clostridium pasteurianum*. Bioresour Technol.

[CR28] Mori H, Shimizu S, Yamane T (1985). Automatic supplementation of minerals in fed-batch culture to high cells mass concentration. Biotechnol Bioenerg.

[CR29] Oskouie SFG, Tabandeh F, Yakhchali B, Eftekhar F (2007). Enhancement of alkaline protease production by *Bacillus clausii* using Taguchi experimental design. Afr J Biotechnol.

[CR30] Pasternak K, Kocot J, Horecka A (2010). Biochemistry of magnesium. J Elementol.

[CR31] Poornejada N, Karimi K, Behzad T (2013). Improvement of saccharification and ethanol production from rice straw by NMMO and [BMIM][OAc] pretreatments. Ind Crop Prod.

[CR32] Qureshi N, Ezeji TC, Ebener J (2008). Butanol production by *Clostridium beijerinckii* part I: use of acid and enzyme hydrolyzed corn fiber. Bioresour Technol.

[CR33] Qureshi N, Saha BC, Hector RE, Hughes SR, Cotta MA (2008). Butanol production from wheat straw by simultaneous saccharification and fermentation using *Clostridium beijerinckii*: part I—batch fermentation. Biomass Bioenerg.

[CR34] Ranjan A, Moholkar VS (2011) Comparative study of various pretreatment techniques for rice straw saccharification for the production of alcoholic biofuels. Fuel. doi:10.1016/j.fuel.2011.03.030

[CR35] Ranjan A, Moholkar VS (2012). Biobutanol: science, engineering and economics. Int J Energ Res.

[CR36] Roberto IC, Mussatto SI, Rodrigues RCLB (2003). Dilute-acid hydrolysis for optimization of xylose recovery from rice straw in a semi-pilot reactor. Ind Crop Prod.

[CR37] Rodrigues L, Moldes A, Teixeira J, Oliveira R (2006). Kinetic study of fermentative biosurfactant production by *Lactobacillus* strains. Biochem Eng J.

[CR38] Sanjari M, Taheri AK, Movahedi MR (2009). An optimization method for radial forging process using ANN and Taguchi method. Int J Adv Manuf Technol.

[CR39] Shukla R, Cheryan M (2001). Zein: the industrial protein from corn. Ind Crop Prod.

[CR40] Singh A, Bishnoi NR (2012). Optimization of ethanol production from microwave alkali pretreated rice straw using statistical experimental designs by *Saccharomyces cerevisiae*. Ind Crop Prod.

[CR41] Sirisansaneeyakul S, Luangpipat T, Vanichsriratana W, Srinophakum T, Chen HHH, Chisti Y (2007). Optimization of lactic acid production by immobilized *Lactococcus lactis* IO-1. J Ind Microbiol Biotechnol.

[CR42] Somda MK, Savadogo A, Barro N, Thonart P, Trarore AS (2011). Effect of mineral salts in fermentation process using mango residues as carbon source for bioethanol production. Asian J Ind Eng.

[CR43] Soni BK, Soucaille P, Goma G (1987). Continuous acetone–butanol fermentation: influence of vitamins on the metabolic activity of *Clostridium acetobutylicum*. Appl Microbiol Biotechnol.

[CR44] Venil CK, Lakshmanaperumalsamy P (2009). Taguchi experimental design for medium optimization for enhanced production by *Bacillus subtilis* HB04. eJST.

[CR45] Virunanon C, Ouephanit C, Burapatana V, Chulalaksananukul W (2013). Cassava pulp enzymatic hydrolysis process as a preliminary step in bio-alcohols production from waste starchy resources. J Clean Prod.

[CR46] Walter KA, Bennett GN, Papoutsakis ET (1992). Molecular characterization of two *Clostridium acetobutylicum* ATCC 824 butanol dehydrogenase isozyme genes. J Bacteriol.

[CR47] Wang X, Wang Y, Wang B, Blaschek HP, Feng H, Li Z (2013). Biobutanol production from fiber-enhanced DDGS pretreated with electrolyzed water. Renew Energy.

[CR48] Wu X, Yang H, Guo L (2010). Effect of operation parameters on anaerobic fermentation using cow dung as a source of microorganisms. Int J Hyd Energ.

[CR49] Xu Y, Hanna MA (2010). Optimum conditions for dilute acid hydrolysis of hemicellulose in dried distillers grains with soluble. Ind Crop Prod.

[CR50] Yu CT, Chen WH, Men LC, Hwang WS (2009). Microscopic structure features changes of rice straw treated by boiled acid solution. Ind Crop Prod.

