# Supplementary material for: Development of semi-defined rice straw-based medium for butanol production and its kinetic study
Source: 3 Biotech. 2013 Mar 9;3(5):353–64. doi: 10.1007/s13205-013-0120-x (PMC3781261; doi:10.1007/s13205-013-0120-x)

**Supplementary Material**

Figure A: Custom fabricated flask for anaerobic culture of *Clostridium acetobutylicum* MTCC 481


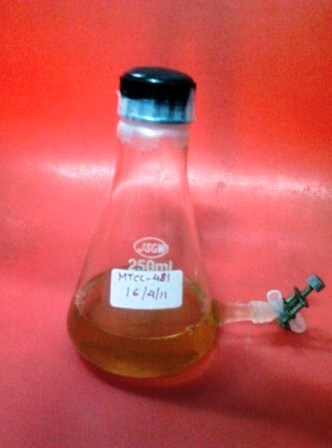

Supplement: Supplementary file 1 — Supplementary material 1 (DOC 50 kb) [file 13205_2013_120_MOESM1_ESM.doc]
